# Is blood pressure reduction a valid surrogate endpoint for stroke prevention? an analysis incorporating a systematic review of randomised controlled trials, a by-trial weighted errors-in-variables regression, the surrogate threshold effect (STE) and the biomarker-surrogacy (BioSurrogate) evaluation schema (BSES)

**DOI:** 10.1186/1471-2288-12-27

**Published:** 2012-03-12

**Authors:** Marissa N Lassere, Kent R Johnson, Michal Schiff, David Rees

**Affiliations:** 1 School of Public Health and Community Medicine, Faculty of Medicine, University of New South Wales, Sydney 2052, NSW, Australia; 2 Department of Rheumatology, St George Hospital, Sydney, NSW 2217, Australia; 3 St. George Clinical School, Faculty of Medicine, University of New South Wales, Sydney 2217, NSW, Australia; 4 Department of Cardiology, St. George Hospital, Sydney 2217, NSW, Australia

**Keywords:** Blood pressure, Stroke, Surrogate Endpoint, Biomarker

## Abstract

**Background:**

Blood pressure is considered to be a leading example of a valid surrogate endpoint. The aims of this study were to (i) formally evaluate systolic and diastolic blood pressure reduction as a surrogate endpoint for stroke prevention and (ii) determine what blood pressure reduction would predict a stroke benefit.

**Methods:**

We identified randomised trials of at least six months duration comparing any pharmacologic anti-hypertensive treatment to placebo or no treatment, and reporting baseline blood pressure, on-trial blood pressure, and fatal and non-fatal stroke. Trials with fewer than five strokes in at least one arm were excluded. Errors-in-variables weighted least squares regression modelled the reduction in stroke as a function of systolic blood pressure reduction and diastolic blood pressure reduction respectively. The lower 95% prediction band was used to determine the minimum systolic blood pressure and diastolic blood pressure difference, the surrogate threshold effect (STE), below which there would be no predicted stroke benefit. The STE was used to generate the surrogate threshold effect proportion (STEP), a surrogacy metric, which with the R-squared trial-level association was used to evaluate blood pressure as a surrogate endpoint for stroke using the Biomarker-Surrogacy Evaluation Schema (BSES3).

**Results:**

In 18 qualifying trials representing all pharmacologic drug classes of antihypertensives, assuming a reliability coefficient of 0.9, the surrogate threshold effect for a stroke benefit was 7.1 mmHg for systolic blood pressure and 2.4 mmHg for diastolic blood pressure. The trial-level association was 0.41 and 0.64 and the STEP was 66% and 78% for systolic and diastolic blood pressure respectively. The STE and STEP were more robust to measurement error in the independent variable than R-squared trial-level associations. Using the BSES3, assuming a reliability coefficient of 0.9, systolic blood pressure was a B + grade and diastolic blood pressure was an A grade surrogate endpoint for stroke prevention. In comparison, using the same stroke data sets, no STEs could be estimated for cardiovascular (CV) mortality or all-cause mortality reduction, although the STE for CV mortality approached 25 mmHg for systolic blood pressure.

**Conclusions:**

In this report we provide the first surrogate threshold effect (STE) values for systolic and diastolic blood pressure. We suggest the STEs have face and content validity, evidenced by the inclusivity of trial populations, subject populations and pharmacologic intervention populations in their calculation. We propose that the STE and STEP metrics offer another method of evaluating the evidence supporting surrogate endpoints. We demonstrate how surrogacy evaluations are strengthened if formally evaluated within specific-context evaluation frameworks using the Biomarker- Surrogate Evaluation Schema (BSES3), and we discuss the implications of our evaluation of blood pressure on other biomarkers and patient-reported instruments in relation to surrogacy metrics and trial design.

## Background

**Figure 1:**
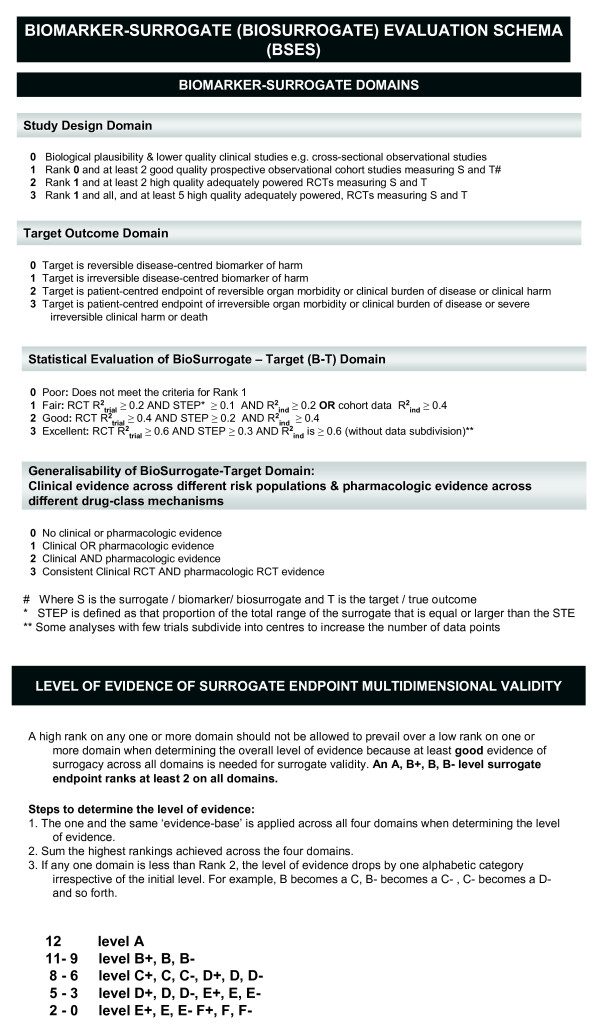
**Biomarker-Surrogacy (BioSurrogate) Evaluation Schema (BSES2011)**.

Substantive discussions of surrogate endpoint validation began in the late 1980s and early 1990s partly driven by the need to find valid biomarkers for Acquired Immunodeficiency Syndrome (AIDS) randomised controlled trials. A systematic review of the literature of statistical methods, conceptual frameworks and schema [1], recently incorporated as Appendix A in the Institute of Medicine's publication Evaluation of Biomarkers and Surrogate Endpoints in Chronic Disease [2], found that statistical validity was a key component of surrogate endpoint evaluation. In this systematic review [1], the 1992 framework by Boissel et al [3], is considered to be the first application of a rigorous multilayered schema for surrogate endpoint evaluation. Boissel's schema proposes that evidence from pathophysiology (biological plausibility), epidemiological studies and randomised controlled trials is needed. Several other frameworks of surrogate validity have been proposed [1,2], including our approach which builds on Boissel's framework. Our schema, designed as an overall and comparative hierarchical multidimensional framework for evaluating biomarkers as surrogates, is the Biomarker-Surrogacy Evaluation Schema (BSES). The BSES1 (also referred to as Quantitative Surrogate Validation Levels of Evidence Schema-QSVLES) published in 2007 [4], had three domains, study design, target outcome and statistical evaluation, as well as add-on penalties which captured concepts of generalisability and risk-benefit. In 2008, the BSES2 populated the statistical domain with specific statistical measures and criteria [1]. In 2010, the BSES3 [5] replaced the penalties with a domain that specifically evaluated clinical and pharmacologic generalisability of the surrogate under evaluation, simplified the number of ranks within each domain, and dropped criteria specific to public health risk-benefit. The BSES3, is a matrix of four domains each with four ranks (see Figure 1 and Additional file 1: Scenarios illustrating the application of the Biomarker-Surrogate (BioSurrogate) Evaluation Schema (BSES3)). It provides a rank for each domain as well as a combined score of surrogacy status. Using the BSES3, the best performing surrogate requires excellent statistical evidence from multiple randomised controlled trials, irreversible morbidity, organ failure or death as the target outcome, and evidence across different drug class mechanisms and clinical risk populations. The BSES3 is data and context driven; therefore, the surrogacy status of a biomarker may change over time as new data and or contexts become available. The statistical domain of the BSES is also informed by and updated to incorporate innovations in statistical methodology.

The *excellent *rank statistical evidence specified in the BSES requires high trial-level association of treatment effects, high individual-level associations and high surrogate threshold effect proportion (STEP) between the surrogate endpoint and the true clinical endpoint. Using mixed model methods, Buyse, Molenberghs [6] and their colleagues [7] proposed and have extensively developed the statistical methodology for trial-level and individual-level associations between surrogate and true clinical endpoints. These associations are coefficients of determination of the trial-level effects of treatment on both endpoints (R^2^_trial_) and of the patient-level association between both endpoints (R^2^_individual_). These statistics provide qualitative and different evaluations of the surrogate endpoint. They are reported as a *relative *measure and can take any value from 0 to 1. The surrogate threshold effect proportion (STEP), proposed by Lassere [1], is also a relative measure, and is derived from its *absolute *measure, the surrogate threshold effect (STE). The STE, developed from work undertaken by Daniel and Hughes [8], and independently proposed by Burzykowski and Buyse [9] and Johnson et al [10,11], provides a method of reporting surrogacy status in the units of the surrogate, for example mmHg of blood pressure. The STE uses a statistical model of past trials that measures both the surrogate and true clinical endpoints to predict the outcome benefit as a function of the surrogate in a new trial that only measures the surrogate endpoint. As the STE is measured in the units of the surrogate, the STE could be used to inform surrogate validity for drug registration and drug reimbursement decisions. The strongest relationships between surrogate and outcome using the STE have been in oncology [10,7]. Here, surrogates such as progression-free survival are being used to predict survival, yet the surrogate itself, a composite of progression and survival, contains the outcome, so some degree of prediction is expected. By contrast, in the case of laboratory biomarker surrogates, there is no such expected relationship. Of laboratory biomarkers, as far as we could determine, only an STE for CD4 cell count as a surrogate for progression to AIDS or death [8] (although not called an STE in this early report) and LDL-cholesterol as a surrogate for cardiovascular mortality have been published [11]. The STE and the STEP provide different qualitative and quantitative statistical information with respect to one another and with respect to trial-level and individual-level measures of association, and we suggest that evidence of surrogacy across several statistical methods is needed to comprehensively inform surrogate decision-making.

Regulators have approved drugs based on surrogate endpoints [12,13]. Blood pressure is a leading example [14]. Blood pressure is a physiological biomarker. The acceptance of blood pressure as a valid surrogate endpoint is supported by evidence from large cohort studies which found that high blood pressure was a risk factor for vascular events [15], and from randomised controlled trials which showed that reduction in blood pressure reduced these events. Additional evidence for the support of blood pressure as a valid surrogate endpoint comes from recent meta-analyses of randomised controlled trials and from meta-regressions [16,18]. The literature on biomarkers and surrogate endpoints consider blood pressure the closest we have to a 'gold standard' surrogate endpoint. If so, the comparative performance of blood pressure on any surrogate evaluation framework is of importance. That the stroke reduction found in randomised controlled trials of hypertension was close to that predicted from epidemiological studies of hypertension [3] was supportive evidence of blood pressure as a valid surrogate for stroke. Yet, there has been no quantitative evaluation of the surrogacy status of blood pressure on any framework and no report has determined a surrogate threshold effect (STE) for blood pressure. These were the two aims of our study in the context of systolic and diastolic blood pressure (BP) as surrogate endpoints of stroke prevention.

## Methods

### Trial inclusion and exclusion criteria

Randomised trial evidence of the relationship of blood pressure reduction and rate of stroke was reviewed from the published literature. Trial inclusion criteria were negatively (placebo or open-label) controlled trials that randomised patients that (i) were at least six months duration of pharmacologic treatment for primary or secondary prevention, (ii) used any anti-hypertensive regimen, (iii) reported baseline and on-trial blood pressure for treated and for control patients, and (iv) reported number of events for fatal and non-fatal stroke for treated and for control patients. A negatively controlled trial was a trial with no mandatory anti-hypertensive treatment requirement for patients in the control arm. However, discretionary treatment, for example, rescue medication, was permitted. Exclusion criteria were (i) trials that only recruited patients with chronic heart failure, diabetes or chronic renal failure, (ii) trials in patients with acute stroke or acute myocardial infarct, (iii) trials designed to assess blood pressure in responders rather than all randomised patients, (iv) trials using a second on-trial randomisation that re-assigned some active arm patients to placebo treatment, (v) trials that simultaneously evaluated multiple interventions targeting vascular risk (e.g. hypertension and hyperlipidaemia) and (vi) trials with fewer than five cerebrovascular (CVA) events per treatment arm [18]. We did not require elevated blood pressure at entry as an inclusion criterion.

### Search strategy

Our first search strategy was a search of Medline (1950 to February 2009) for randomised trials of blood pressure using exp Hypertension/(174,452) OR exp Blood Pressure/(210,864) OR (blood pressure or hyperten* or systolic or diastolic).mp. (523,114) that identified 528,263 citations. These were limited to (humans AND clinical trial, all OR clinical trial, phase I OR clinical trial, phase II OR clinical trial, phase III OR clinical trial or controlled clinical trial OR multicentre study OR randomized controlled trial) yielding 50,780 unique citations, a random selection of which identified most as unsuitable for our objective and a systematic evaluation of all was not feasible. Therefore, an alternative strategy to identify trials that met our inclusion criteria was applied.

Our second strategy was a rapid review process to identify original trial reports that met our trial inclusion criteria by sourcing secondary data identified in meta-analyses of randomised trials of hypertension. We searched Medline (1950 to May 2009) using the search terms (exp Hypertension/(181,231) OR exp Blood Pressure/(216,603) OR (blood pressure or hyperten* or systolic or diastolic).mp. (538,084); limited to humans AND adults AND to meta-analysis OR reviews OR systematic reviews. This yielded 1199 citations. A search of the Cochrane Database identified 8 reviews that already had been identified in the Medline search. The abstracts of these 1199 citations were evaluated and 38 meta-analyses or systematic reviews reported trials that satisfied our inclusion criteria (see Additional file 2 reference list). The remaining meta-analyses did not contribute trials because they were meta-analyses of trials that (i) were of insufficient duration, (ii) did not address adult patients with hypertension, (iii) were derivative analyses of earlier meta-analyses with no new trials, or they were not meta-analyses at all. All randomised controlled trial reports identified from these 38 meta-analyses were obtained to determine whether they met our inclusion/exclusion criteria. One author (KJ) further hand-searched all citations of the randomised controlled trial reports to identify trials that may have been missed by the 38 meta-analyses.

### Data extraction

Two authors (KJ, MS) independently extracted the data from trial reports and uncertainty was further adjudicated (ML). Intention-to-treat extractions were applied throughout. We extracted the following for treated and control patients: treatments at trial entry; initial, on-trial and final diastolic and systolic blood pressure; fatal and non-fatal stroke events (CVAs), and any other interventions used. We also extracted all cardiovascular fatal events and all-cause fatal events if the trial reported fatal and non-fatal stroke events. Trials enrolling both primary and secondary prevention patients were coded as combined primary and secondary prevention. We also extracted, if reported, information on trial: demographics (age, gender), additional risk factors (e.g. smoking history, diabetes), trial year, trial size, trial duration, trial blinding, and the proportion of subjects that were blood pressure treatment naïve, had past treatment or were on current treatment at trial entry. We also recorded whether add-on treatment was permitted in either arm if protocol defined blood pressure targets were not met and the proportion that required that add-on treatment. No publication included individual level data for analysis.

### Data analysis

The surrogate endpoint independent variables were two, diastolic blood pressure and systolic blood pressure. Each was a difference between arms of the differences over the trial for each arm. For diastolic blood pressure (DBP) the difference over the trial was defined as the mean baseline DBP in the treated patients minus the mean on-trial DBP (i.e., all measures from year 1 to end-of-trial) in the treated patients, and similarly for the control patients. The DBP independent variable was then the mean DBP difference in treated patients minus the mean DBP difference in control patients. The systolic blood pressure independent variable was defined similarly. Systolic and diastolic blood pressure differences were analysed separately. The dependent variable was the relative risk reduction (RRR) of fatal and non-fatal stroke, i.e., the stroke rate in the control arm minus the stroke rate in the test arm, divided by the stroke rate in the control arm. RRR was selected as the most intuitively understandable outcome metric for clinicians. All trial data are from intention-to-treat analysis. If a trial report pre-specified a comparison combining treatment arms, these were used. Otherwise, the comparison used from multiple arm trials was the comparison with the largest difference in BP changes. We used the BSES3 for the multidimensional quantitative evaluation of blood pressure reduction as a surrogate endpoint for stroke events.

### Statistical analysis

The independent variable mean blood pressure difference is an estimated variable; therefore, its true value is not known with certainty in regression analysis. When both the independent variable as well as the dependent variable are measured with error, then the effect of the independent variable is biased, usually towards the null (underestimated) [19,20]. There are several errors-in-variables regression methods that have been proposed to adjust for this bias [20]. One method adjusts for the bias by incorporating knowledge of the reliability, r, (where r = 1- (noise variance/total variance)) of the independent variable. Therefore we undertook a weighted (by trial size) errors-in-variables regression of relative risk reduction (RRR) of fatal and non-fatal stroke on systolic and diastolic blood pressure reduction respectively, incorporating sensitivity analyses with reliabilities of 0.6, 0.7, 0.8 and 0.9, where 1.0 indicates no measurement error. The weighting was applied to (i) estimates of the linear prediction, (ii) the standard error of the predicted expected value and (iii) the standard error of the point prediction for a single observation, commonly referred to as the standard error of the future or forecast value. We used data from the literature to inform these reliabilities as there have been many studies that report within and between individual, as well as within and between group variability of systolic and diastolic blood pressure, plus specific studies of sources of variability including measurement error of blood pressure observations. We used Tobit regression (assuming no uncertainty in the estimated surrogate) [21] because relative risk reduction is bounded at 1.0, representing 100% increase. We also used fractional polynomial regression [22] to confirm that a linear model was satisfactory. Other regression assumptions were evaluated using standard methods. The surrogate threshold effect (STE) is the minimum by-arm blood pressure reduction difference that predicts a by-arm stroke reduction benefit. Graphically, this is where the regression lower 95% prediction line [23] for an individual trial crosses the horizontal axis representing no stroke reduction benefit [9]. Other statistics reported are the R-squared at the trial level (R^2^_trial-level_) of the weighted errors-in-variables regression model as well as the coefficient (slope) of the blood pressure reduction difference. No publication included individual level data therefore, we were unable to determine the patient-level association between both endpoints (R^2^_individual_).

Given that reduction of blood pressure with antihypertensive drugs has a greater effect on stroke prevention than on reduction of cardiovascular mortality or on all-cause mortality [24,25], we evaluated the construct validity of the STE by repeating all the statistical analyses with cardiovascular deaths and all-cause deaths as the patient-relevant clinical endpoints. We used Stata 11 for all analyses.

## Results

**Figure 2:**
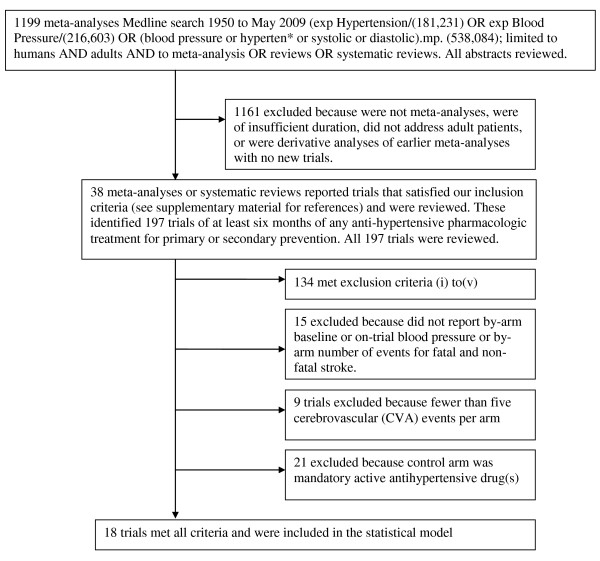
**Flow chart of articles.** (also see Table 1 and Additional file 1).

The search of the 38 meta-analyses yielded 197 individual trials that were greater than 6 months duration and were of any anti-hypertensive pharmacologic treatment. Hand-searching of the citations from these trial reports did not identify any trials missed by the 38 meta-analyses. All 197 trials including all secondary reports and publications were reviewed and data extracted. Of these 63 were for at least six months of pharmacologic treatment for primary or secondary prevention and were not excluded based on exclusion criteria (i) to (v). Of these 48 reported pre- and on-trial blood pressure and cerebrovascular events. Of these, 39 had at least 5 cerebrovascular events in each arm. Of these, 18 were negatively controlled [26-43] (see Figure 2 flow chart); all but one, HYVET-pilot [39], used placebo and were blinded. Trial, clinical and pharmacologic characteristics of these 18 trials are described in Table 1. One trial, ANBP1[26], reported only DBP differences. Trial size, BP reductions, and stroke event numbers are shown in Table 2.

**Table 1:** Trial, clinical and pharmacologic characteristics of the 18 trials that met inclusion criteria

Trial	Year	Trial Size	Mean Trial Duration (yrs)	Prevention	Drug (first agent/s)	Add on Drug (s)#	Drug Class*	Mean SBP* at base- line	Mean DBP* at base- line	Mean Age	Male %	Diabetes %	Smoker %
ANBP1 [26]	1980	3427	4	Primary	chlorothiazide	N	0,1,2	157.4	100.5	50.4	63.0	0.0	25.0

IPPPSH [27]	1985	6357	4	Mixed	Oxprenolol	Y	1,2	173.0	107.5	52.2	50.2		29.1

MRC-mild [28]	1985	17354	5	Mixed	bendrofluazide or propranolol	N	1,2	161.5	98.5	50.0	50.0		31.0

HEP [29]	1986	884	8	Primary	Atenolol	N	0,1,2	196.4	98.9	68.8	30.9	0.0	24.3

SHEP [30]	1991	4736	4.5	Primary	Chlorthalidone	N	1,2	170.3	76.6	71.6	43.2	10.1	12.7

STOP [31]	1991	1627	2.1	Primary	atenolol or hydrochlorothiazide + amiloride or metoprolol or pindolol	N	1,2	195.0	102.0	75.7	37.0		8.0

MRC-elderly [32]	1992	4396	5.8	Primary	atenolol or amiloride/ hydrochlorothiazide	N	1,2	184.7	90.7	70.3	41.8	0.0	17.5

PATS [33]	1995	5665	2	Secondary	Indapamide	N	1	153.8	92.8	60.0	72.0		

Syst-Eur [34]	1997	4695	2	Primary	Nitrendipine	N	1,3,4	173.9	85.5	70.3	33.2		7.3

PREVENT [35]	2000	825	3	Secondary	Amlodipine	Y	2,3,4	129.4	78.9	56.9	80.1		24.7

PROGRESS [36]	2001	6105	4	Secondary	Perindopril	N	1,3	147.0	86.0	64.0	70.0	12.5	20.0

EUROPA [37]	2003	12218	4.2	Secondary	Perindopril	Y	1,2,3,4	137.0	82.0	60.0	85.4	12.3	

SCOPE [38]	2003	4937	3.7	Primary	Candesartan	Y	1,2,3,4,5	166.3	90.4	76.4	35.5	12.0	8.7

HYVET-pilot [39]	2003	857	1.1	Primary	bendroflumethiazide or lisinopril	N	1,3,4	181.5	99.6	83.8	36.6		4.2

PEACE [40]	2004	8290	4.8	Secondary	Trandolapril	Y	1,2,3,4	133.5	78.0	64.0	82.0	17.0	14.5

ACTION [41]	2004	7665	4.9	Secondary	Nifedipine	Y	1,2,3,4	137.5	79.9	63.4	79.4	14.5	17.7

TRANSCEND [42]	2008	5926	4.7	Secondary	Telmisartan	Y	1,2,4,5	141.0	81.9	66.9	57.0	35.7	9.8

HYVET-main [43]	2008	3845	2.1	Primary	Indapamide	N	1,4	173.0	90.8	83.6	39.5	6.8	6.5

# Possible anti-hypertensive pharmacologic treatment at entry or add-on during trial to any trial arm. *Drug Class: 0 = older drugs (methyldopa, clonidine etc), 1 = diuretics, 2 = beta-blockers 3 = angiotensin converting enzyme (ACE) inhibitors, 4 = calcium channel blockers (CCBs), 5 = angiotensin receptor blockers (ARBs, also called angiotensin II receptor antagonists (AIIRA))

**Table 2:** Trial size, blood pressure difference (mean change in active arm minus mean change in control arm), stroke events and stroke relative risk reduction

Trial	Year	**No**. Subject s In Active Arm	**No**. **Of** Subjec ts In Contro l Arm	Systolic Blood Pressure Difference	Diastolic Blood Pressure Difference	Stroke events in Active Arm	Stroke events in Control Arm	Stroke Relative Risk Reduction
ANBP1 [26]	1980	1721	1706	*NR*	5.7	13	22	0.41

IPPPSH [27]	1985	3185	3172	3.8	0.2	45	46	0.03

MRC-mild [28]	1985	8700	8654	10.3	6.2	60	109	0.45

HEP [29]	1986	419	465	18.0	11.0	23	44	0.42

SHEP [30]	1991	2365	2371	12.4	4.3	106	163	0.35

STOP [31]	1991	812	815	22.5	10.5	29	53	0.45

MRC-elderly [32]	1992	2183	2213	14.3	7.2	101	134	0.24

PATS [33]	1995	2824	2841	5.9	3.0	159	217	0.26

Syst-Eur [34]	1997	2398	2297	9.4	5.5	49	80	0.41

PREVENT [35]	2000	417	408	6.8	3.9	5	5	0.02

PROGRESS [36]	2001	3051	3054	9.0	4.0	317	420	0.24

EUROPA [37]	2003	6110	6108	5.0	2.0	98	102	0.04

SCOPE [38]	2003	2477	2460	3.5	2.9	89	115	0.23

HYVET-pilot [39]	2003	431	426	23.9	11.1	12	18	0.34

PEACE [40]	2004	4158	4132	3.0	1.2	71	92	0.23

ACTION [41]	2004	3825	3840	4.7	3.1	82	108	0.24

TRANSCEND [42]	2008	2954	2972	3.4	2.0	112	136	0.17

HYVET-main [43]	2008	1933	1912	12.0	6.0	51	69	0.27

*NR *= Not Reported

The initial treatment agent included a diuretic in 8 trials, a beta-blocker in 5 trials, a calcium channel blockers in 3 trials, an angiotensin converting enzyme (ACE) inhibitor in 4 trials and an angiotensin II receptor antagonists in 2 trials. Only one trial, PATS [33], limited treatment to a single pharmacologic class. Ten trials included treatment from three or more pharmacologic classes. Seven of the 18 trials included a variable proportion of patients on diuretics, beta-blockers, calcium channel blockers or ACE inhibitors, either at study entry (background therapy) or as add-on during the trial. Six of these 7 were trials published in the last 10 years. In only 4 trials (ANBP1[26], MRC-mild[28], HEP[29], MRC-elderly [32]) were all subjects antihypertensive treatment naïve at trial onset. Nine trials were conducted in the primary prevention setting. Mean trial duration was 3.9 years. Only one trial was less than two years in duration (HYVET-pilot [39] with a mean duration of treatment of 1.1 years) and 11 were four or more years. Mean age of subjects across the 18 trials was 66 years and 55% were male. Mean blood pressure at trial entry was 160 mmHg systolic and 90 mmHg diastolic.

**Figure 3:**
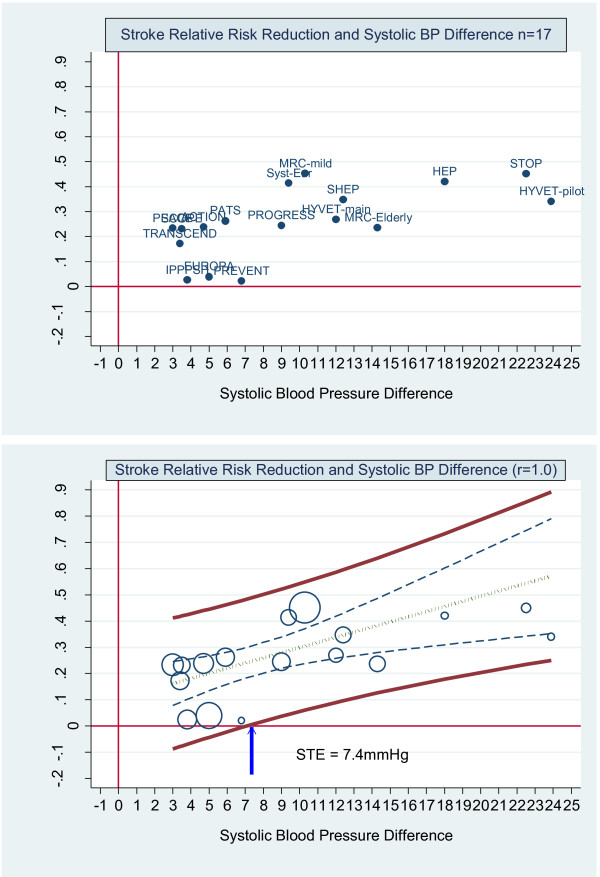
****Stroke relative risk reduction and systolic BP difference - no measurement error. **Upper graph:** Scatterplot of stroke relative risk reduction and systolic blood pressure difference reduction showing the 17 trials labelled by trial name. **Lower graph:** scatterplot weighted by trial size and by trial weighted least squares regression of stroke relative risk and systolic blood pressure difference reduction assuming no measurement error (reliability coefficient = 1.0). The upper and lower bold solid lines are the upper and lower 95% prediction limits, the dashed inner lines are the 95% confidence limits, the dot-dash centre line is the mean regression line. The arrow indicates where the lower 95% prediction line intersects with the x axis. This is the Surrogate Threshold Effect (STE) for stroke reduction and is the systolic blood pressure difference needed to impute a stroke reduction benefit in a new trial.

The by-trial scatterplot of stroke relative risk and systolic blood pressure difference labelled by trial name is shown in upper graph of Figure 3. The lower graph in Figure 3 shows the same scatterplot weighted by trial size and the by-trial weighted least squares regression of stroke relative risk and systolic blood pressure difference assuming no uncertainty in the estimated systolic blood pressure difference. The upper and lower bold solid lines are the upper and lower 95% prediction limits, the dashed inner lines are the 95% confidence limits, the dot-dash centre line is the mean regression line. The arrow indicates where the lower 95% prediction line intersects the horizontal axis at approximately 7.4 mmHg. This is the surrogate threshold effect (STE). This means that a future trial would need a SBP difference, active versus control, of at least 7.4 mmHg to ensure a stroke reduction benefit. The slope of the regression line is positive at 0.02 (*p *< 0.01) and the R^2 ^was 0.37.

**Figure 4:**
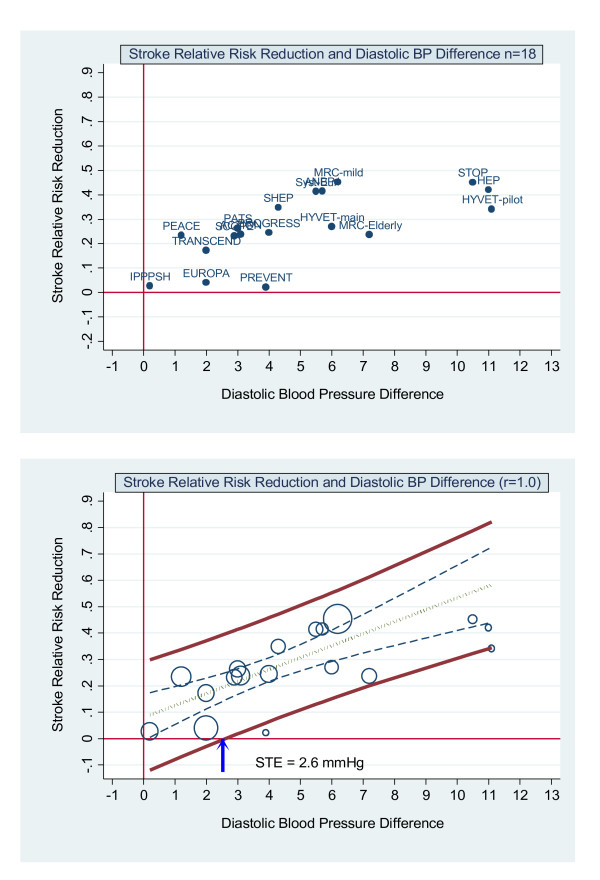
**Stroke relative risk reduction and diastolic BP difference - no measurement error. Upper graph**: Scatterplot of stroke relative risk reduction and diastolic blood pressure difference reduction showing the 18 trials labelled by trial name. **Lower graph**: scatterplot weighted by trial size and by trial weighted least squares regression of stroke relative risk and systolic blood pressure difference reduction assuming no measurement error (reliability coefficient = 1.0). The upper and lower bold solid lines are the upper and lower 95% prediction limits, the dashed inner lines are the 95% confidence limits, the dot-dash centre line is the mean regression line. The arrow indicates where the lower 95% prediction line intersects with the x axis. This is the Surrogate Threshold Effect (STE) for stroke reduction and is the diastolic blood pressure difference needed to impute a stroke reduction benefit in a new trial.

The results for diastolic blood pressure are in Figure 4. The surrogate threshold effect is 2.6 mmHg. The slope of the regression line is positive at 0.045 (*p *< 0.001) and the R^2 ^is 0.58.

**Figure 5:**
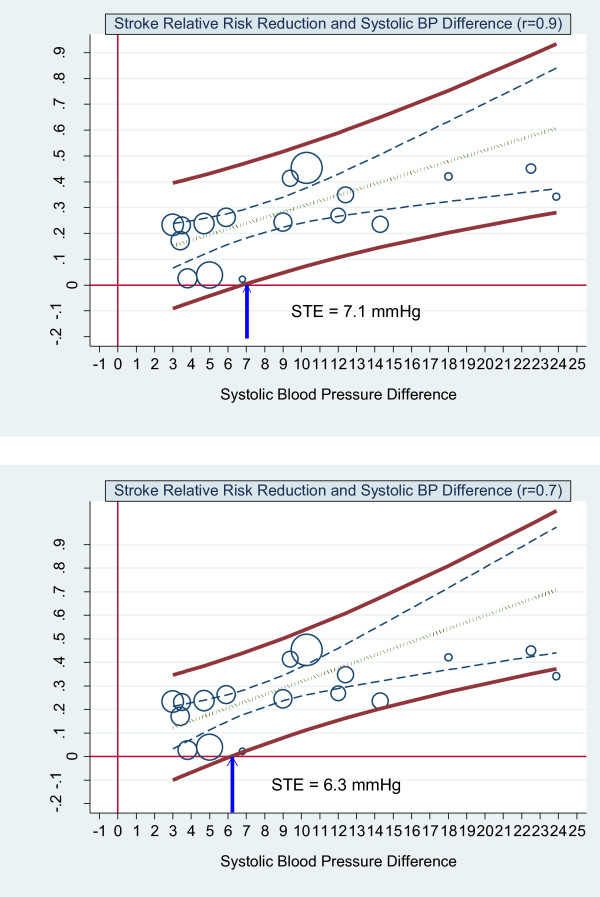
**Stroke relative risk reduction and systolic BP difference - errors-in-variables. Upper graph**: scatterplot weighted by trial size and by trial errors-in-variables (eiv) weighted least squares regression of stroke relative risk and systolic blood pressure difference reduction assuming measurement error (reliability coefficient = 0.9). **Lower graph**: scatterplot weighted by trial size and by trial eiv weighted least squares regression of stroke relative risk and systolic blood pressure difference reduction assuming measurement error (reliability coefficient = 0.7). The upper and lower bold solid lines are the upper and lower 95% prediction limits, the dashed inner lines are the 95% confidence limits, the dot-dash centre line is the mean regression line. The arrow indicates where the lower 95% prediction line intersects with the x axis. This is the Surrogate Threshold Effect (STE) for stroke reduction and is the systolic blood pressure difference needed to impute a stroke reduction benefit in a new trial given eiv regression.

**Figure 6:**
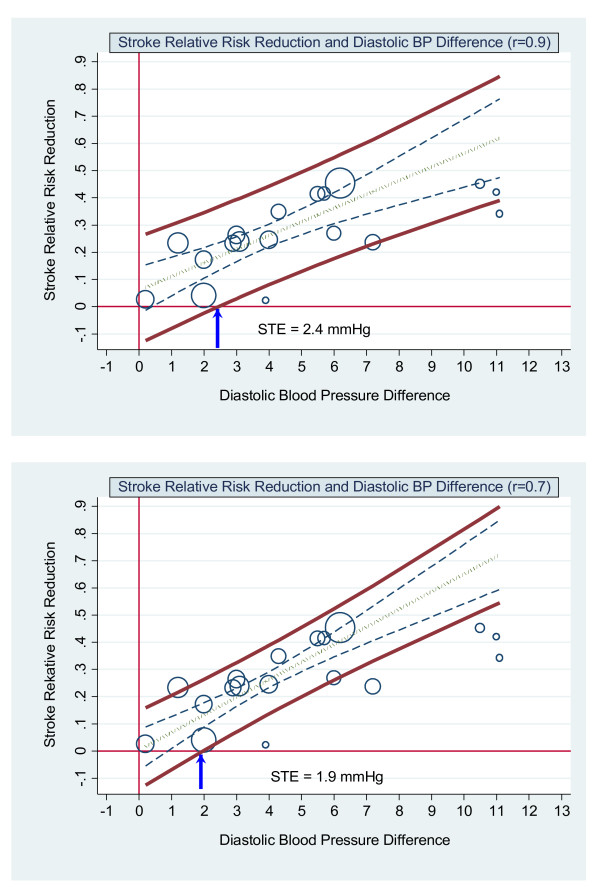
**Stroke relative risk reduction and diastolic BP difference - errors-in-variables. Upper graph**: scatterplot weighted by trial size and by trial errors-in-variables (eiv) weighted least squares regression of stroke relative risk and diastolic blood pressure difference reduction assuming measurement error (reliability coefficient = 0.9). **Lower graph**: scatterplot weighted by trial size and by trial eiv weighted least squares regression of stroke relative risk and diastolic blood pressure difference reduction assuming measurement error (reliability coefficient = 0.7). The upper and lower bold solid lines are the upper and lower 95% prediction limits, the dashed inner lines are the 95% confidence limits, the dot-dash centre line is the mean regression line. The arrow indicates where the lower 95% prediction line intersects with the x axis. This is the Surrogate Threshold Effect (STE) for stroke reduction and is the diastolic blood pressure difference needed to impute a stroke reduction benefit in a new trial given eiv regression.

Baseline blood pressure was not a significant coefficient. Table 3 shows the results for the errors-in-variables regression with 0.9, 0.8, 0.7 and 0.6 reliability coefficients for both systolic and diastolic blood pressure respectively. These results include the slope (coefficient of systolic and diastolic mean blood pressure reduction), the p-value for the slope, the R-squared of the linear regression model, the STE and the STEP. The slope and R-squared of the linear regression model increase as the reliability coefficient decreases. This in turn decreases the STE and increases the STEP. Figure 5 shows the graphs of the scatterplot weighted by trial size and the by-trial weighted least squares regression of stroke relative risk and systolic blood pressure difference assuming reliability coefficient of 0.9 and 0.7. Figure 6 shows the results for diastolic blood pressure for these same reliability coefficients.

**Table 3:** Weighted ordinary least squares errors-in-variables regression of stroke relative risk reduction and systolic and diastolic blood pressure difference respectively, assuming 0.9, 0.8, 0.7 and 0.6 reliability coefficients for estimated blood pressure difference

Reliability coefficient	Regression Slope	Regression Slope p-value	R^2^	STE*	STEP**
	**Systolic Blood Pressure**				

**1.0**	0.0196	0.010	0.37	7.4	64%

**0.9**	0.0217	0.008	0.41	7.1	66%

**0.8**	0.0245	0.006	0.46	6.7	68%

**0.7**	0.0280	0.004	0.52	6.3	70%

**0.6**	0.0327	0.002	0.61	5.9	72%

					

		**Diastolic Blood Pressure**			

**1.0**	0.0453	0.000	0.58	2.6	76%

**0.9**	0.0503	0.000	0.64	2.4	78%

**0.8**	0.0566	0.000	0.72	2.2	80%

**0.7**	0.0647	0.000	0.83	1.9	83%

**0.6**	0.0755	0.000	0.96	1.2	88%

					

* Surrogate Threshold Effect ** Surrogate Threshold Effect Proportion

**Figure 7:**
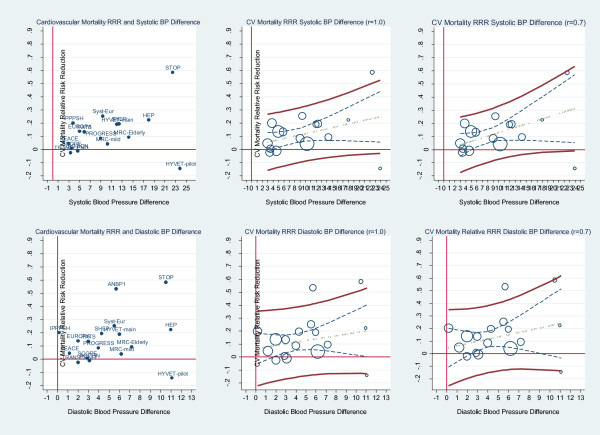
**Cardiovascular mortality relative risk reduction and BP difference.Upper graphs**: Left: Scatterplot of cardiovascular mortality (CVM) relative risk reduction (RRR) and systolic blood pressure difference reduction showing the trials labelled by trial name. Middle: scatterplot weighted by trial size and by trial weighted least squares regression of cardiovascular mortality relative risk reduction and systolic blood pressure difference reduction assuming no measurement error (reliability coefficient = 1.0). Right: scatterplot weighted by trial size and by trial weighted least squares regression of cardiovascular mortality relative risk reduction and systolic blood pressure difference reduction assuming measurement error (reliability coefficient = 0.7). **Lower graphs**: show the results for diastolic blood pressure. The upper and lower bold solid lines are the upper and lower 95% prediction limits, the dashed inner lines are the 95% confidence limits, the dot-dash centre line is the mean regression line. There is no Surrogate Threshold Effect (STE) to impute a CV mortality benefit in a new trial.

In contrast to stroke, no STE could predict a cardiovascular mortality benefit. Assuming a reliability coefficient of 1.0 (i.e. no measurement error in the surrogate) for systolic blood pressure, the slope of the mean regression line is 0.009 and is non-significant, the R-square is 0.15, the STE approaches the x axis at 25 mmHg, but it does not cross the axis; therefore, there is no STE and no STEP. In diastolic blood pressure the slope is 0.012 and is also non-significant, the R-squared is only 0.05 and the STE approaches no value of diastolic blood pressure reduction within the model data-points. These results are displayed in Figure 7. The upper 3 graphs are the results for systolic blood pressure and the lower 3 graphs for diastolic blood pressure. The left-side graph shows the scatterplot weighted by trial size and labelled by the trial name. The middle and right-side graphs show the weighted least squares regression of cardiovascular mortality relative risk assuming reliability coefficient of 1.0 and 0.7.

**Figure 8:**
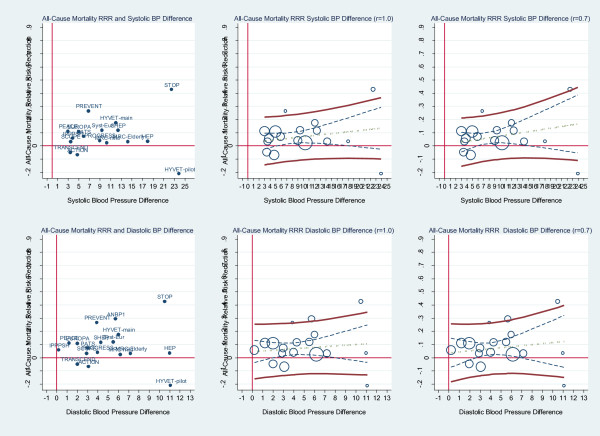
**All-cause mortality relative risk reduction and BP difference. Upper graphs**: Left: Scatterplot of all-cause mortality (ACM) relative risk reduction (RRR) and systolic blood pressure difference reduction showing the trials labelled by trial name. Middle: scatterplot weighted by trial size and by trial weighted least squares regression of all-cause mortality relative risk reduction and systolic blood pressure difference reduction assuming no measurement error (reliability coefficient = 1.0). Right: scatterplot weighted by trial size and by trial weighted least squares regression of all-cause mortality relative risk reduction and systolic blood pressure difference reduction assuming measurement error (reliability coefficient = 0.7). **Lower graphs**: show the results for diastolic blood pressure. The upper and lower bold solid lines are the upper and lower 95% prediction limits, the dashed inner lines are the 95% confidence limits, the dot-dash centre line is the mean regression line. There is no Surrogate Threshold Effect (STE) to impute an all-cause mortality benefit in a new trial.

Similarly, no STE predicts an all-cause mortality benefit. Assuming a reliability coefficient of 1.0 (i.e. no measurement error in the surrogate) for systolic blood pressure, the slope is 0.005 and is non-significant, the R-squared is 0.06, the STE approaches infinity. In diastolic blood pressure the slope is 0.005 and is also non-significant, the R-squared is only 0.02 and there is no STE. These results are displayed in Figure 8.

The performance of blood pressure as a surrogate endpoint for stroke events according to the BSES3 is reported in Table 4. As we had no patient-level data, we could not derive the R^2^_individual_ specific to our dataset, therefore we assumed that the R^2^_individual_ was as least as large as the R^2^_trial_. Under assumptions of *minimal *uncertainty (reliability coefficient 0.9) systolic blood pressure reduction is a B + surrogate endpoint for stroke prevention as there is good statistical evidence from multiple (n = 17) randomised controlled trials, of irreversible morbidity (stroke), across different drug class mechanisms (at least 5) and clinical risk populations (gender, age, ethnicity and primary/secondary prevention). Under assumptions of uncertainty (reliability coefficient 0.9) diastolic blood pressure is an A grade surrogate endpoint for stroke prevention, because it scored the highest rank on all domains including the statistical domain (excellent statistical evidence). However, under the assumption of no uncertainty, (reliability coefficient 1.0), both systolic and diastolic blood pressure performed less well because they dropped rank on the statistical domain. The BSES3 requires a valid surrogate endpoint to have a combined score of at least 9 (one domain of rank 3 and the remainder at least of rank 2) and a minimum threshold rank of at least 2 across all domains (see Figure 1). This design prevents a high score on any one or more domain compensating for a low score on one or more domain; because good evidence of surrogacy across all domains is needed to be a valid surrogate endpoint. If the threshold criterion is not met, the grade drops by one alphabetic grade. Systolic blood pressure fell from rank 2 to rank 1 in the statistical domain. As a result, systolic blood pressure no longer met the criteria of requiring a minimum rank of 2 across all domains. Although the combined score was 10, the grade dropped from B to C. Diastolic blood pressure fell from rank 3 to rank 2 on the statistical domain. Its combined score was 11, but because it met the minimum rank of 2 across all domains, it held its grade of B +.

**Table 4:** The surrogacy status of blood pressure reduction for stroke prevention on the BSES3 assuming a reliability coefficient of r = 1.0 (no uncertainty) and assuming a reliability coefficient of r=0.9 (minimal uncertainty)

	Study Design	Target Outcome	*Statistical* *Relationship* Note1	Generalisability	Combined	Grade
Systolic r = 1.0	3	3	***1***	3	10	**C** **See Note** **2**

Systolic r = 0.9	3	3	*2*	3	11	B+

Diastolic r = 1.0	3	3	*2*	3	11	B+

Diastolic r = 0.9	3	3	*3*	3	12	A

Note 1: We did not have individual level data from any randomised controlled trial needed to evaluate individual-level associations therefore we assumed that the R_individual_^2 ^was as least as large as the R^2^_trial_

Note 2: Although the combined score is 10, the statistical relationship was less than rank 2, therefore the grade drops from B to C

## Discussion

Using trial-level data from published negatively controlled randomised trials of anti-hypertensive drugs we formally evaluated the evidence that supports systolic and diastolic blood pressure reduction as a surrogate endpoint for stroke reduction. We also determined the STE for systolic and diastolic blood pressure, i.e., the minimum systolic and diastolic blood pressure difference needed in a new trial to predict a stroke benefit. Using errors-in-variables regression weighted by trial size, and assuming minimal uncertainty (reliability coefficient 0.9) estimating trial-level systolic and diastolic blood pressure reduction, the STE for systolic blood pressure is 7.1 mmHg and the STE for diastolic blood pressure is 2.4 mmHg. Furthermore, assuming having patient-level data would not influence the results on the BSES3, systolic blood pressure is a Grade B + surrogate endpoint for stroke protection and diastolic blood pressure is a Grade A surrogate endpoint for stroke protection. A discussion of the assumptions that underpin these results, supporting evidence, and caveats are fundamental to the debate on surrogate endpoint evaluation specific to blood pressure as well as to other biomarkers and patient-reported instruments.

### Context of surrogacy and impact of secular change in study design, trial populations and treatment modalities

These STEs for systolic and diastolic blood pressure assume that the new trial measuring only the surrogate endpoint is otherwise similar to past trials used in the predictive model regarding intervention, population, trial design and pharmacologic therapy. These STEs were derived from negatively controlled antihypertensive randomised controlled trials of varying design, clinical populations and pharmacologic classes. Therefore, these STEs may not be applicable to trials that require subjects in all arms to be randomised to an anti-hypertensive drug, or to trials of homogeneous populations, for example, trials of only heart or renal failure patients. As our data-set of trials spans four decades, secular changes in standard of care of hypertension is to be expected and is reflected in changes in trial design and in relation to inclusion and exclusion criteria of risk populations. At one extreme, severe hypertension rapidly became incompatible with trials with no treatment controls. Another secular change is the large number of different pharmacologic classes of agents used singly and in combination. As the evidence for the benefits of blood pressure reduction accrued, equipoise for negatively controlled trials diminished, and as more pharmacologic classes of anti-hypertensive agents appeared, polypharmacy became the rule. Few patients were treatment-naïve, and trials increasingly used designs with provisions for add-on to established "background" regimens. Trial entry criteria changed as did the cut-off for mandatory discontinuation in the event of the on-trial development of severe, uncontrolled hypertension. Older patients and those with lower entry BP and improved risk profiles generally were increasingly enrolled in more recent trials. In seven trials, a proportion of enrolled patients (sometimes up to half) were already on antihypertensive drugs either for hypertension or other indications. We did not exclude these trials because they met our inclusion criteria. Also, to capture drug effects that may occur with BP lowering from a baseline high-normal range, we did not require elevated BP at baseline. Cross-over also occurred in some trials, although generally it was less than 15% and occurred from active to control arm as well as in the other direction. Only four trials recruited treatment-naïve patients exclusively, where one might expect greater BP and outcome responses compared to trials with treatment-experienced patients. Fourteen trials had varying proportions of treatment-naïve versus treatment-experienced patients which also might dilute or delay the effect on stroke reduction. These changes are likely to attenuate the relationship between blood pressure and stroke, impede the compilation of new data supporting surrogacy, and increase the size of the STE. It is likely that the STEs estimated from active-control randomised trials would be larger because subjects in all arms are randomised to hypertensive drugs. Furthermore, trials clinically of homogeneous populations, such as heart or renal failure populations may also have different STEs. These hypotheses, if substantiated, imply that the initial evaluation of all existing and new biomarkers should be in negatively controlled heterogeneous randomised trials; otherwise, the surrogacy potential of these biomarkers may be underestimated.

### Regression modelling with measurement error in the independent variable: Application to blood pressure

Ordinary linear regression has several assumptions, one being that all variation is in the dependent variable and that as long as these measurement errors are uncorrelated and unbiased the results are not influenced. However, the independent variable must be measured without error. In the presence of random measurement error in the independent variable coefficients are biased towards the null. In our analysis both stroke relative risk reduction and mean blood pressure reduction are trial-level estimations. Therefore, a priori, they are measured with error. Unfortunately, trials did not report the within arm standard deviation of blood pressure reduction. Individual blood pressure readings are highly variable, because of position, rest, etc, and measurement errors due to the instrument and observer [44,45]. Systematic bias, such as white coat hypertension [46], is generally less problematic in the setting of a randomised controlled trial. Hebel [47] evaluated within-person variability of diastolic BP. Within occasion variability was 3.1 mmHg for patients on medication and 2.4 mmHg for normotensive controls. Reliability coefficients of 0.6 to 0.9 have been reported [48-52]. Skirton [53] recently undertook a systematic review of variability and reliability of manual and automated blood pressure readings, but none of the results were reported as reliability coefficients. The importance of blood pressure variability as a predictor of vascular events is a new direction of research [54]. The underestimation of risk association due to regression dilution in long-term prospective studies has also been well described in studies of hypertension [55,56]. Therefore, our linear regression model is influenced by several sources of variation all of which, assuming no systematic variation, underestimate the slope of the relationship between stroke relative risk reduction and blood pressure reduction. Therefore, our results are conservative, and we illustrate the effect of adjusting for the error in the independent variable by sensitivity analysis using several reliability coefficients. Using errors-in-variables regression systolic STEs vary from 7.4 mmHg (assuming no uncertainty around the estimated effects on the independent variable) to 5.9 (assuming a reliability coefficient as low as 0.6). The diastolic STEs vary from 2.6 mmHg (assuming no uncertainty around the estimated effects on the independent variable) to as little as1.2 (again assuming a reliability coefficient as low as 0.6). Interestingly, the difference in STEs across these different reliability coefficients was only 1.5 and 1.4 mmHg for systolic and diastolic blood pressure respectively, a indicator of the robustness of STE and the STEP as a method of evaluating surrogacy. There was a much greater impact of different reliability coefficients on the R-squared trial-level association. These almost doubled for both systolic and diastolic blood pressure (R-squared 0.37 to 0.61 and 0.58 to 0.96) with increasing correction for measurement error. We used the reliability coefficient errors-in-variables regression (as provided by Stata statistical software), however, several other methods have been proposed [19,20] and comparing the results of different methods would be worthy of further research.

### Statistical measures of surrogacy

Surrogacy is a complex construct and requires several qualitatively different statistical (and substantive) metrics for its evaluation. We were limited to the STE, the STEP and trial-level linear regression to statistically evaluate blood pressure's surrogacy status. We did not have access to any patient-level data, therefore, were unable to evaluate surrogacy status on an individual-level. Other approaches have been proposed, including a mixed models analysis [7] that combine time, surrogate, clinical endpoint, treatment, trial and individual subject variables into a single analysis for estimation of a mixed model trial-level association, a mixed model individual-level association and a mixed model STE. Other new methods are those based on principal stratification [57]. The STEP is useful because it identifies the relative position of the STE within the model of data used to determine the STE by converting a unit-specific STE, e.g. mmHg of blood pressure, to a proportion. It is similar to the coefficient of determinations in that it can take a value from 0.0 to 1.0 (or 0% to 100%). The STEP serves to compare STEs across different contexts. We have already shown that the STE is more robust to measurement error than the R-squared trial-level association; therefore, the STEP may also be a more robust method of comparing different surrogate endpoints.

Our trial-level association for systolic blood pressure may be considered low (R-squared 0.37 assuming no uncertainty). The results for diastolic blood pressure were somewhat better (R-squared 0.58 assuming no uncertainty). Trial-level associations of reported surrogate endpoints that are considerably higher (> 0.8) are those that evaluate progression-free survival, disease-free survival and event-free survival as a surrogate endpoint for overall survival, where survival is included in the surrogate [58-60]. Analyses of time to progression or response rate surrogate endpoints, measures that do not include a survival in the surrogate, report more modest trial-level associations. In metastatic colon disease the trial-level association of time to progression was 0.33 [10]. Biomarkers in non-oncological chronic disease also have trial-level associations that are modest. In negatively-controlled randomised trials of statins the trial-level association of LDL-cholesterol reduction and cardiovascular mortality reduction assuming no uncertainty was 0.41 [11].

### Previously published models and meta-analyses of stroke and other vascular outcomes

Others have found similar relationships using different regression methods relating BP differences and expected outcome differences, but none calculated prediction bands to estimate an STE. Staessen et al [16] was the first to publish a blood pressure trial level regression. Using data from hypertension outcome trials of at least 2-years duration and at least 100 patients, they reported trial-level regression models of the odds ratio of cardiovascular mortality and all cardiovascular events versus systolic blood pressure difference. Graphically, a systolic BP difference of 5 mmHg corresponded to a cardiovascular mortality reduction of about 10%. In our data-set of trials, designed to identify stroke outcomes, a systolic blood pressure reduction of 10 mmg was associated with a 9% reduction of cardiovascular mortality, but the result was not statistically significant. Law et al [18] published a meta-analysis of stroke in 45 trials (including trials in conditions other than essential hypertension) demonstrating a 5 mmHg diastolic blood pressure or 10 mmHg SBP reduction corresponded to a 41% reduction in stroke. However, among the 45 trials were studies in patients with coronary heart disease that reported no BP data at all, and for these trials BP changes were imputed from results from an earlier study of short term BP studies [61]. We found, in our trial-dataset, a 5 mmHg DBP reduction or 10 mmHg SBP reduction corresponded to a 22.5% and 20% reduction in stroke respectively, assuming a no measurement error model. Boissel et al [62] in the INDANA data set that included individual patient data reported a Cox proportional hazard model of stroke in the five trials (28,997 patients, 808 events). They found a hazard ratio for stroke of 0.79, a risk reduction of 21%. In this study about one-half of the benefit was accounted for when adding an adjustment made for on-treatment BP and BP measurement error, a result interpreted by the authors as suggesting that only half of the stroke benefit is "explained by" the effect of treatment on blood pressure. We found that baseline blood pressure was not a significant predictor of stroke events, a result confirmed by others [63].

Others have also found that the relationship for stroke reduction is stronger than that for cardiovascular mortality or all-cause mortality reduction [24]. We therefore would expect the STE for mortality reduction to be larger than the STE for stroke reduction. All trials in our data reported all-cause fatal events and all but one (PREVENT [35]) reported cardiovascular fatal events. Trial-level associations for both systolic and diastolic blood pressure reduction and cardiovascular mortality and all-cause mortality relative risk reduction were extremely poor, less than 0.15 for systolic and 0.05 for diastolic blood pressure, assuming no measurement uncertainty. We were surprised to find that the relationship between blood pressure reduction and mortality reduction in our stroke dataset was too poor for an STE to be estimated. In our stroke data-set, there was no blood pressure reduction that predicted a mortality benefit. Although these findings provide additional support for the validity of the stroke-specific STEs, we cannot conclude that there is no STE for mortality reduction. Our dataset a required at least 5 stroke outcomes per treatment arm. To determine the STEs for cardiovascular mortality and all-cause mortality, a search strategy specific to this research question is needed. A less conservative statistical analysis using mixed models and individual-level data may also prove necessary before we conclude that blood pressure is not a valid surrogate endpoint for mortality endpoints.

### Potential limitations and further qualifications of the STE and other conclusions

Are there any caveats that may bias the STE and our conclusions towards non-conservative results? We propose that the STE, STEP and trial-level associations we have reported are conservative. As we only had access to trial level data we could not undertake a full hierarchical mixed model regression. Our model could also be vulnerable to ecological bias. Our preliminary unpublished simulation work in SAS on comparing ordinary least squares (OLS) regression with a joint hierarchical linear mixed model has indicated that OLS regression STE is almost always larger therefore conservative compared to the STE obtained from the linear mixed model. In fact, the exceptional simulation scenarios seem to be clinically highly implausible ones; for example, models with very few (5) trials, very small (50 patients) trials, or very small between-trial outcome variance compared to the patient-level outcome variance. Furthermore, these scenarios presented computational problems when fitting a linear mixed model, while the by-trial model is computationally straightforward. In fact, because of its ability to incorporate both trial and patient-level variances, the mixed model may be expected to produce narrower prediction bands.

Our simulation work also suggests that the OLS STE is not influenced by individual-level correlation between surrogate and true endpoints. Therefore, the STE may be less subject to ecological bias. Individual-level correlation is usually first explored in observation cohort studies, as was the case in blood pressure, and is often the basis for subsequent evaluation in randomised controlled trials.

Other potential methodological weaknesses that may bias the STE towards a non-conservative value deserve mention. Even though some individual patient analyses suggest the hazard ratio for stroke varies over time [64], we assume the hazard is constant; individual patient data are needed to study this question. Incorporating patient-level variability could increase the STE, but, to date, we have not found that patient-level variability is as great an influence on the STE as number of trials and between-trial outcome variance in our simulations. Nevertheless, patient-level data would facilitate greater exploration of differences in trial populations over time and may generate more precise context-specific STEs. Although expecting that all patient level data on all trials to be available through publication is unrealistic, analysis informed by a incorporating a random subset of patient level data may prove useful and deserves further investigation. Another issue we considered is whether it is necessary to calculate a confidence interval around the STE. This was discussed at a recent international workshop on biomarkers as surrogate endpoints (Sydney Bio-Surrogates Workshop, February 2011, unpublished), and most workshop participants did not think it appropriate to calculate a confidence interval around a prediction interval.

### Concepts to define surrogacy evaluation and qualification past, present and future: Lessons learned from the evaluation of blood pressure

It is important that the concepts that define surrogate validity are developed in a properly conceived context. Formal statistical, rather than anecdotal, evaluation of rigorously defined populations and other context-determined heterogeneity is needed to systematically explore and compare surrogacy status of biomarkers and patient-reported instruments. Our results support that diastolic blood pressure reduction is a very good surrogate for stroke prevention. Systolic blood pressure performs less well. Our analysis of mortality endpoints indicates that the surrogacy of blood pressure is more context specific than generally appreciated. The context could be drug-class specific, clinical endpoint specific and clinical population specific (i.e., by age, gender, ethnicity and comorbidity). How much is the effect on cardiovascular mortality mediated by blood pressure reduction, and how much by other drug class effects? Once the clinical endpoint is broadened to all-cause mortality, the effect of blood pressure is further diluted by other drug class effects, including drug class toxicity. Recently it has been shown that different drug classes mediate a heterogeneous effect on stroke through variable effects of blood pressure visit-to-visit variability [65] and visit-to-visit variability is an independent risk factor for vascular events. It is likely that risk predictions that include a composite surrogate endpoint of mean blood pressure reduction and reduced blood pressure variability and instability might estimate different STEs and provide stronger evidence of surrogacy for blood pressure [66] across a variety of clinical endpoints.

Using the BSES the surrogacy status of different biomarkers and patient-reported instruments can be compared within these specified contexts. The STE, a trial-level metric, is a useful measure because it provides information on the surrogate endpoint in the units of the surrogate and therefore could be used to inform drug registration and drug reimbursement decisions that are based on surrogate endpoints. Moreover, the STE is needed to determine the STEP, which we have shown is a robust relative metric of surrogacy. We should emphasise that we have not analysed data nor do we report a metric that provide decisions regarding individual patients, for example, how much to decrease an individual patient DBP or what is the optimal final DBP target to use. For those claims to be data-driven would require, for example, an RCT to demonstrate that lowering DBP to, say, 80 mmHg is superior (fewer strokes) than lowering the DBP to 85 mmHg. Undertaking such 'target' trials is feasible but difficult as evidenced by the experience of the Hypertension Optimal Treatment (HOT) Study [67,68]. The epidemiology of BP seems to indicate that even very small BP differences result in difference in vascular event rates, so, in theory, trials could be designed to demonstrate a benefit from even very small BP differences. Policy recommendations would then need to be made on a numbers-needed-to-treat basis and, of course, integrated into the other risk factors for any given patient [69]. These issues are quite distinct from the strength of evidence of a surrogate, the aim of the STE and BSES.

Formal evaluation of surrogacy status using a standardised framework, such as the BSES3, can be used to begin discussions on a surrogate biomarkers qualification process [2]. However, surrogacy applications in the real world of patient-care or policy decisions take into account other factors that are not included in the BSES3. These factors of risk-benefit such as public health, drug safety, disease rarity or diseases that are serious or life-threatening and where there are no alternative therapies, do not directly impact the internal validity of surrogacy but nevertheless are important considerations for regulators and payors [12]. Nonetheless, the BSES3 provides clinicians and others a simple hierarchical framework that can now be used for critically appraising studies of biomarkers and surrogate endpoints.

## Conclusions

In this report we provide the first surrogate threshold effect (STE) values for systolic and diastolic blood pressure. The STEs appear to have face and content validity as evidenced by the inclusivity of trial populations, subject populations and pharmacologic intervention populations in their calculation. The STE and STEP metrics offer another method of evaluating the evidence supporting surrogate endpoints. We have demonstrated how surrogacy evaluations are strengthened if formally evaluated within specific-context evaluation frameworks using the Biomarker- Surrogate Evaluation Schema (BSES3), and we note the implications of our evaluation of blood pressure on other biomarkers and patient-reported instruments in relation to surrogacy metrics and trial design.

## Abbreviations

STE: Surrogate threshold effect; BP: Blood pressure; SBP: Systolic blood pressure; DBP: Diastolic blood pressure; RRR: Relative risk reduction; RCT: Randomised controlled trial; CVA: Cerebrovascular accident; ACE: Angiotensin converting enzyme; ARB: Angiotensin II receptor blocker.

## Competing interests

The authors declare that they have no competing interests.

## Authors' contributions

ML contributed to the conception, design and co-ordination of the study, carried out duplicate data extraction for some randomised controlled trial articles and items, compiled the results, performed the statistical analysis and co-drafted the manuscript. KJ contributed to the conception and design, carried out data extraction for all meta-analysis and randomised controlled trial articles and items, compiled the results, and co-drafted the manuscript. MS performed the Medline searches, carried out duplicate data extraction for some randomised controlled trial articles and data items, and commented on the manuscript. DR contributed to the design, and commented on the manuscript. All authors read and approved the final manuscript.

## Pre-publication history

The pre-publication history for this paper can be accessed here:

http://www.biomedcentral.com/1471-2288/12/27/prepub

## Supplementary Material

Additional file 1 **Appendix 1**. Scenarios illustrating the application of the Biomarker-Surrogate (BioSurrogate) Evaluation Schema (BSES3). Click here for file

Additional file 2 **Reference list of 38 meta-analyses or systematic reviews reporting trials that satisfied our inclusion criteria**. Click here for file
