# Supplementary material for: Is blood pressure reduction a valid surrogate endpoint for stroke prevention? an analysis incorporating a systematic review of randomised controlled trials, a by-trial weighted errors-in-variables regression, the surrogate threshold effect (STE) and the biomarker-surrogacy (BioSurrogate) evaluation schema (BSES)
Source: BMC Med Res Methodol. 2012 Mar 12;12:27. doi: 10.1186/1471-2288-12-27 (PMC3388460; doi:10.1186/1471-2288-12-27)
Supplement: Additional file 1 — Appendix 1. Scenarios illustrating the application of the Biomarker-Surrogate (BioSurrogate) Evaluation Schema (BSES3). [file 1471-2288-12-27-S1.DOC]

**Supplementary Material 1. Scenarios illustrating the application of the Biomarker-Surrogate (BioSurrogate) Evaluation Schema (BSES3)**

- The BSES level of evidence is determined by summing the highest rankings achieved across the four domains. The one and the same ‘evidence-base’ is applied across all four domains when determining the level of evidence. However, a high rank on any one or more domain should not be allowed to prevail over a low rank on one or more domain when determining the overall level of evidence because at least good evidence of surrogacy across all domains is needed for surrogate validity. Therefore, the BSES also incorporates an additional constraint to ensure that the level of evidence for a surrogate endpoint has hierarchical multidimensional validity: if any one domain is less than rank 2, the level of evidence drops by one alphabetic category irrespective of the initial level. For example, B becomes a C, B- becomes a C- , C- becomes a D- and so forth.
- **An A, B+, B, B- level surrogate endpoint ranks at least 2 on all domains.**
  - **This is illustrated by the following scenarios.**
    - **Scenario 1.** Biomarker BZ has been evaluated in all (and at least 5) high quality randomised trials and at least 2 cohort studies therefore Study Design Domain is rank 3. In all these studies the target outcome was death therefore Target Outcome Domain is rank 3. Furthermore, statistical metrics of these studies are good because R^2^_trial_ ≥ 0.4 AND STEP ≥ 0.2 AND R^2^_individual_ ≥ 0.4, therefore Statistical Evaluation Domain is rank 2. However the above RCT data is limited to only one pharmacologic drug class therefore Generalisability Domain cannot be rank 3.
      - Scenario 1(i). If there are good cohort data supporting evidence of pharmacologic, demographic and clinical group generalisability, the Generalisability Domain is rank 2. Therefore the combined score is 3+3+2+2 = 10, which would be B level of evidence if all four domains are at least rank 2. They are all at least rank 2 therefore Biomarker X is a B level of evidence.
      - Scenario 1(ii). If there are **no** cohort data of pharmacologic, demographic and clinical group generalisability, then the Generalisability Domain is only rank 1. The combined score3+3+2+1= 9 which would be B- level of evidence if all four domains are at least rank 2. As all domains are not at least 2, Biomarker X level of evidence drops from B- to C- level of evidence.
    - **Scenario 2.** Biomarker BY has been evaluated in all (and at least 5) high quality randomised trials and at least 2 cohort studies (Study Design Domain rank 3), where the target outcome was death (Target Outcome Domain rank 3), the statistical metrics are only fair R^2^_trial_ ≥ 0.2 AND STEP ≥ 0.1 AND R^2^_individual_ ≥ 0.2, (Statistical Evaluation Domain is rank 1) and the RCTs are from all the many different pharmacologic drug classes, and demographic and clinical groups (Generalisability domain is rank 3).
      - The combined score is 3+3+1+3=10, which would be B level of evidence if all four domains are at least rank 2, but all domains not at least 2, therefore the alphabetic grade drops from B to C level of evidence.

- - - **Scenario 3.** Biomarker BX has been evaluated in all (and at least 5) high quality randomised trials and at least 2 cohort studies (Study Design Domain rank 3), where the target outcome was stable angina (Target Outcome Domain rank 2), and the statistical metrics are good – R^2^_trial_ ≥ 0.4 AND STEP ≥ 0.2 AND R^2^_individual_ ≥ 0.4, (Statistical Evaluation Domain is rank 2)and the RCTs are from all the many different pharmacologic drug classes, and demographic and clinical groups (Generalisability domain is rank 3).
      - The combined score 3+2+2+3=10, all domains at least 2, B level of evidence.
    - **Scenario 4.** Biomarker BW has been evaluated in no randomised controlled trials but in at least 2 cohort studies (Study Design Domain rank 1), where the target outcome was stable angina (Target Outcome Domain rank 2), and the statistical metrics are fair. There is no RCT data but the cohort data R^2^_ind_ ≥ 0.4, (Statistical Evaluation Domain is rank 1) and the cohort studies are from all the many different pharmacologic drug classes, and demographic and clinical groups (Generalisability domain is rank 2).
      - The combined score 1+2+1+2=6, C- level of evidence, but as two of the domains are not at least rank 2, the alphabetic grade drops to D- level of evidence.
    - **Scenario 5**. Biomarker BV has been evaluated in 3 cross-sectional studies (Study Design Domain rank 0), where the target outcome was cardiovascular mortality (Target Outcome Domain rank 3), and the statistical metrics are poor. There is no RCT data and no prospective longitudinal cohort data (Statistical Evaluation Domain is rank 0) and the cross-sectional studies are from all pharmacologic drug classes, and demographic and clinical groups (Generalisability domain is rank 2).
      - The combined score 0+3+0+2=5, D+ level of evidence, but as two of the domains are not at least rank 2, the alphabetic grade drops to E+ level of evidence.
    - **Scenario 6.** Biomarker BU has been evaluated in 3 cross-sectional studies (Study Design Domain rank 0), where the target outcome was coronary calcification on CT (Target Outcome Domain rank 1), and the statistical metrics are poor. There is no RCT data and no prospective longitudinal cohort data (Statistical Evaluation Domain is rank 0) and the cross-sectional studies are from all pharmacologic drug classes, and demographic and clinical groups (Generalisability domain is rank 2).
      - The combined score 0+1+0+2=3, D- level of evidence, but as three of the domains are not at least rank 2, the alphabetic grade drops to E- level of evidence.
    - **Scenario 7**. Biomarker BT has been evaluated in 3 cross-sectional studies (Study Design Domain rank 0), where the target outcome was coronary calcification on CT (Target Outcome Domain rank 1), and the statistical metrics are poor. There is no RCT data and no prospective longitudinal cohort data (Statistical Evaluation Domain is rank 0) and the cross-sectional studies are limited to one pharmacologic drug class, and to healthy adults less than 65 years (Generalisability domain is rank 0).
      - The combined score 0+1+0+0=1, E+ level of evidence, all domains are not at least rank 2, therefore alphabetic grade drops to F+ level of evidence.
    - **Scenario 8.** Biomarker BS has been evaluated in 3 cross-sectional studies (Study Design Domain rank 0), where the target outcome was serum BNP (Target Outcome Domain rank 0), and the statistical metrics are poor. There is no RCT data and no prospective longitudinal cohort data (Statistical Evaluation Domain is rank 0) and the cross-sectional studies are limited to one pharmacologic drug class, and to healthy adults less than 65 years (Generalisability domain is rank 0).
      - The combined score 0+0+0+0=0, E- level of evidence, all domains are not at least rank 2, therefore alphabetic grade drops to F- level of evidence.
