# Supplementary material for: Is blood pressure reduction a valid surrogate endpoint for stroke prevention? an analysis incorporating a systematic review of randomised controlled trials, a by-trial weighted errors-in-variables regression, the surrogate threshold effect (STE) and the biomarker-surrogacy (BioSurrogate) evaluation schema (BSES)
Source: BMC Med Res Methodol. 2012 Mar 12;12:27. doi: 10.1186/1471-2288-12-27 (PMC3388460; doi:10.1186/1471-2288-12-27)
Supplement: Additional file 2 — Reference list of 38 meta-analyses or systematic reviews reporting trials that satisfied our inclusion criteria. [file 1471-2288-12-27-S2.DOC]

**Supplementary Material 2:** Reference list of 38 meta-analyses and systematic reviews (from a search strategy that identified 1199 citations and abstracts) reviewed by KRJ. From these 38 articles KRJ identified 197 randomised controlled trials that possibly met the criteria for the meta-regression.

1.	Staessen JA, Wang JG, Thijs L. Cardiovascular protection and blood pressure reduction: a meta-analysis. Lancet 2001; 358(9290):1305-15.

2.	Blood Pressure Lowering Treatment Trialists' Collaboration, Turnbull F, Neal B *et al*. Blood pressure-dependent and independent effects of agents that inhibit the renin-angiotensin system. J Hypertens 2007; 25(5):951-8.

3.	Yusuf S, Peto R, Lewis J, Collins R, Sleight P. Beta blockade during and after myocardial infarction: an overview of the randomized trials. Prog Cardiovasc Dis 1985; 27(5):335-71.

4.	MacMahon SW, Cutler JA, Furberg CD, Payne GH. The effects of drug treatment for hypertension on morbidity and mortality from cardiovascular disease: a review of randomized controlled trials. Prog Cardiovasc Dis 1986; 29 (3 Suppl 1):99-118.

5.	Collins R, Peto R, MacMahon S *et al*. Blood pressure, stroke, and coronary heart disease. Part 2, Short-term reductions in blood pressure: overview of randomised drug trials in their epidemiological context. Lancet 1990; 335(8693):827-38.

6.	Law MR, Wald NJ, Morris JK, Jordan RE. Value of low dose combination treatment with blood pressure lowering drugs: analysis of 354 randomised trials. BMJ 2003; 326(7404):1427.

7.	Wald DS, Law M, Morris JK, Bestwick JP, Wald NJ. Combination therapy versus monotherapy in reducing blood pressure: meta-analysis on 11,000 participants from 42 trials. Am J Med 2009; 122(3):290-300.

8.	Law MR, Morris JK, Wald NJ. Use of blood pressure lowering drugs in the prevention of cardiovascular disease: meta-analysis of 147 randomised trials in the context of expectations from prospective epidemiological studies. BMJ 2009; 338:b1665.

9.	Neal B, MacMahon S, Chapman N, Blood Pressure Lowering Treatment Trialists' Collaboration. Effects of ACE inhibitors, calcium antagonists, and other blood-pressure-lowering drugs: results of prospectively designed overviews of randomised trials. Blood Pressure Lowering Treatment Trialists' Collaboration. Lancet 2000; 356(9246):1955-64.

10.	Turnbull F, Blood Pressure Lowering Treatment Trialists' Collaboration. Effects of different blood-pressure-lowering regimens on major cardiovascular events: results of prospectively-designed overviews of randomised trials. Lancet 2003; 362(9395):1527-35.

11.	Blood Pressure Lowering Treatment Trialists' Collaboration, Turnbull F, Neal B *et al*. Effects of different regimens to lower blood pressure on major cardiovascular events in older and younger adults: meta-analysis of randomised trials. BMJ 2008; 336(7653):1121-3.

12.	Thijs L, Fagard R, Lijnen P, Staessen J, Van Hoof R, Amery A. A meta-analysis of outcome trials in elderly hypertensives. J Hypertens 1992; 10(10):1103-9.

13.	Staessen JA, Wang JG, Thijs L. Calcium-channel blockade and cardiovascular prognosis: recent evidence from clinical outcome trials. Am J Hypertens 2002; 15(7 Pt 2):85S-93S.

14.	Staessen JA, Wang JG, Thijs L. Cardiovascular prevention and blood pressure reduction: a quantitative overview updated until 1 March 2003. J Hypertens 2003; 21(6):1055-76.

15.	Staessen JA, Wang JG, Thijs L. What can be expected from optimal blood pressure control? J Hypertens Suppl 2003; 21(2):S3-9.

16.	Angeli F, Verdecchia P, Reboldi GP *et al*. Calcium channel blockade to prevent stroke in hypertension: a meta-analysis of 13 studies with 103,793 subjects. Am J Hypertens 2004; 17(9):817-22.

17.	Gueyffier F, Boutitie F, Boissel JP *et al*. Effect of antihypertensive drug treatment on cardiovascular outcomes in women and men. A meta-analysis of individual patient data from randomized, controlled trials. The INDANA Investigators. Ann Intern Med 1997; 126(10):761-7.

18.	Lievre M, Gueyffier F, Ekbom T *et al*. Efficacy of diuretics and beta-blockers in diabetic hypertensive patients. Results from a meta-analysis. The INDANA Steering Committee. Diabetes Care 2000; 23 Suppl 2:B65-71.

19.	Boutitie F, Gueyffier F, Pocock S, Fagard R, Boissel JP, INDANA Project Steering Committee. INdividual Data ANalysis of Antihypertensive intervention. J-shaped relationship between blood pressure and mortality in hypertensive patients: new insights from a meta-analysis of individual-patient data. Ann Intern Med 2002; 136(6):438-48.

20.	Boissel JP, Gueyffier F, Boutitie F, Pocock S, Fagard R. Apparent effect on blood pressure is only partly responsible for the risk reduction due to antihypertensive treatments. Fundamental & Clinical Pharmacology 2005; 19(5):579-84.

21.	Wiysonge CS, Bradley H, Mayosi BM *et al*. Beta-blockers for hypertension.Cochrane Database of Systematic Reviews 2007; (1):CD002003.

22.	Heran BS, Wong MM, Heran IK, Wright JM. Blood pressure lowering efficacy of angiotensin converting enzyme (ACE) inhibitors for primary hypertension. Cochrane Database of Systematic Reviews 2008; (4):CD003823.

23.	Heran BS, Wong MM, Heran IK, Wright JM. Blood pressure lowering efficacy of angiotensin receptor blockers for primary hypertension. Cochrane Database of Systematic Reviews 2008; (4):CD003822.

24.	Musini VM, Fortin PM, Bassett K, Wright JM. Blood pressure lowering efficacy of renin inhibitors for primary hypertension. Cochrane Database of Systematic Reviews 2008; (4):CD007066.

25.	Mulrow C, Lau J, Cornell J, Brand M. Pharmacotherapy for hypertension in the elderly. [Review] [46 refs]. Cochrane Database of Systematic Reviews 2000; (2):CD000028.

26.	Quan A, Kerlikowske K, Gueyffier F, Boissel JP, INDANA investigators. Pharmacotherapy for hypertension in women of different races. . Cochrane Database of Systematic Reviews 2000; (3):CD002146.

27.	Lip GY, Makin AJ. Treatment of hypertension in peripheral arterial disease. [Review] [69 refs]. Cochrane Database of Systematic Reviews 2003; (4):CD003075.

28.	Arguedas JA, Perez MI, Wright JM. Treatment blood pressure targets for hypertension. [Review] [64 refs]. Cochrane Database of Systematic Reviews 2009; (3):CD004349.

29.	Lechat P, Packer M, Chalon S, Cucherat M, Arab T, Boissel JP. Clinical effects of beta-adrenergic blockade in chronic heart failure: a meta-analysis of double-blind, placebo-controlled, randomized trials.[see comment]. Circulation 1998; 98(12):1184-91.

30.	Flather MD, Yusuf S, Kober L *et al*. Long-term ACE-inhibitor therapy in patients with heart failure or left-ventricular dysfunction: a systematic overview of data from individual patients. ACE-Inhibitor Myocardial Infarction Collaborative Group. Lancet 2000; 355(9215):1575-81.

31.	Brewster LM, van Montfrans GA, Kleijnen J. Systematic review: antihypertensive drug therapy in black patients.[see comment]. Ann Intern Med 2004; 141(8):614-27.

32.	Doulton TW, He FJ, MacGregor GA. Systematic review of combined angiotensin-converting enzyme inhibition and angiotensin receptor blockade in hypertension. Hypertension 2005; 45(5):880-6.

33.	Lindholm LH, Carlberg B, Samuelsson O. Should beta blockers remain first choice in the treatment of primary hypertension? A meta-analysis. Lancet 2005; 366(9496):1545-53.

34.	Zanchetti A, Grassi G, Mancia G. When should antihypertensive drug treatment be initiated and to what levels should systolic blood pressure be lowered? A critical reappraisal. J Hypertens 2009; 27(5):923-34.

35.	Psaty BM, Lumley T, Furberg CD *et al*. Health outcomes associated with various antihypertensive therapies used as first-line agents: a network meta-analysis. JAMA 2003; 289(19):2534-44.

36.	Wang JG, Staessen JA, Li Y *et al*. Carotid intima-media thickness and antihypertensive treatment: a meta-analysis of randomized controlled trials. Stroke 2006; 37(7):1933-40.

37.	Baguet JP, Legallicier B, Auquier P, Robitail S. Updated meta-analytical approach to the efficacy of antihypertensive drugs in reducing blood pressure. Clinical Drug Investigation 2007; 27(11):735-53.

38.	Heerspink HJ , Ninomiya T, Zoungas S *et al*. Effect of lowering blood pressure on cardiovascular events and mortality in patients on dialysis: a systematic review and meta-analysis of randomised controlled trials. Lancet 2009; 373(9668):1009-15.
